# Looking Back at Undergraduate Research Experiences to Promote the Engagement of Undergraduates in Publishable Research at an R2 Institution

**DOI:** 10.3389/fpsyg.2019.01316

**Published:** 2019-06-04

**Authors:** Jeanine L. M. Skorinko

**Affiliations:** Social Science and Policy Studies Department, Worcester Polytechnic Institute, Worcester, MA, United States

**Keywords:** undergraduate, research, mentor, diversity & inclusion, publish and perish

By the end of my undergraduate career, I worked on six research projects, presented some of this work, and prepared two manuscripts for publication (Hebl and Skorinko, 2005; Skorinko et al., 2006). More importantly, my early engagement in research instilled in me an intellectual curiosity that I had not previously experienced. I started to question everything, and I devoted time to think critically about what I was reading and learning. My love of research grew daily and ultimately changed my career trajectory. Now, as a professor, I find myself drawing upon these experiences, as well as best practices, to develop strategies to conduct publishable research with undergraduate students.

Before delving into the specifics, it is important to set up the institution and program that I am in because this influences the strategies I utilize. My institution is a small-medium private polytechnic institution that has recently moved from an R3 to an R2 by the Carnegie Classification of Institutions of Higher Education. It has a distinct project-based curriculum that allows undergraduates to participate in a unique study abroad program that centers on cross-cultural research experiences. High quality research (including funding) and high quality teaching (including project advising) are expected for tenure and promotion.

Psychological Science is an undergraduate only program within a Social Science department. While Psychological Science tends to be a popular major (Princeton Review, 2018), at my institution it is a smaller, albeit growing, major/minor program. All majors complete a senior thesis. I teach and work with: majors, double majors, minors, those curious about psychology, and those needing a social science requirement. There are benefits (e.g., institutional norm of conducting research with undergraduates), but also challenges (e.g., small number of majors) when engaging undergraduates in publishable research.

Given these institutional parameters, my undergraduate experiences, and my role as faculty, I utilize nine strategies to engage undergraduates in publishable research:

1. **Mindset That All Research Is Publishable**. By adopting the mindset that all research is publishable, I am more engaged and invested in each project—whether my idea or a student's idea. Since I rely on undergraduate research assistants, this mindset is vital.
2. **Enthusiasm**. Research shows that undergraduates relish faculty that are enthusiastic (and approachable) about their empirical investigations (Komarraju et al., 2010; Roberts and Seaman, 2018). As an undergraduate, I gravitated toward faculty who were excited about the work they were conducting. Therefore, I let my enthusiasm for research come out to attract undergraduates to work with me.
3. **Engage Early/Research First Approach**. I engaged in research during my first year as an undergraduate. I had no prior experience, but this was not a hindrance because I was motivated and invested in the project so I wanted to learn methodology and statistics along the way (Pacquiao, 2007). I conducted an ethnographic study that taught me the art of interviewing. I also received a healthy dose of realism about what it takes to conduct qualitative research. The oral histories were presented formally to the community and published in a local magazine. Most importantly, I was hooked. I found a love of asking questions, thinking deeply about topics, and I wanted to keep conducting research. Later on, I discovered that my different research experiences helped me learn methods and statistics *better* because I had real examples I could apply what I was learning to.
  Since early engagement was so important to my undergraduate career, I take a research first approach. If a student wants to learn about research, they can join my lab, regardless of their year or prior experience. I teach them what they need to know along the way. I also use this approach in the classroom. Students conduct small research projects to test theories and practice different methodologies (e.g., observation study, interviews, surveys, or mini-experiments). Student testimonials support early engagement, and others echo the benefits of early engagement as well (Detweiler-Bedell and Detweiler-Bedell, 2019; Dutta et al., 2019).
4. **Recruit Diverse Students**. As an undergraduate, I worked in a diverse lab where different perspectives regarding the research were discussed and incorporated into the projects. I saw firsthand how these novel ideas strengthened the work we were doing. Valuing this, I run a lab whose members are diverse in year in school, experience, academic interests, ethnic background, gender, sexual orientation, etc. There are challenges to managing a diverse lab as it requires flexibility and time (Brew and Mantai, 2017; Peifer, 2019). For example, some students may need to engage in paid work (in the lab or elsewhere), some may struggle academically, and some may not feel like they belong. However, the benefits for the students and the research are worth it. Diverse students who engage in research develop stronger mentoring relationships, feel less isolated, learn to think critically, and are more likely to pursue graduate school (Chan, 2019; Frohardt, 2019; Peifer, 2019; Ahmad et al., under review). By incorporating diverse minds into the process, the research conducted becomes stronger and more inclusive.
5. **Meet Students Where They Are**. When joining the lab, I try to match students on two components: (1) interest in a particular project, and (2) skills they want to learn. This stems from my own experiences because for my first project I simply wanted to gain experience, but after that I had a better understanding of what else I wanted to learn. With more experiences, my confidence and desire to tackle a project on my own grew, and in my senior year I conducted two separate publishable experiments (Hebl and Skorinko, 2005; Skorinko et al., 2006). I find many first-time research assistants want to get their feet wet, but with time and more experience they crave something more. So, I try to have projects in all phases, so I can match students' interests and skills. We also encourage undergraduates to rotate and/or work in multiple labs. I have found that meeting students where they are gets them hooked and keeps them coming back.
6. **Set Realistic Expectations**. We seek different collaborators based on their strengths, and we should do the same with undergraduates. As an undergraduate, I had time to learn programs that my mentor did not have time to learn. For instance, I learned video editing for one project and how to write code that allowed us to put studies online and recruit a non-student population (this was before Survey Monkey, Qualtrics, and MTurk). But, I needed help synthesizing the literature, conducting statistical analyses, and dealing with reviewers. I have to remind myself that an undergraduate does not have the same knowledge or motivation that I do for a project. Therefore, I need to set realistic expectations. For instance, I cannot expect students to know the intricate nuances of the theories as I do; rather, I have to help them develop those insights. Likewise, I cannot expect them to know how to analyze data, but after working through an example, I can have them apply that knowledge to a different research question. To help articulate expectations, some have had success with lab learning agreements/syllabi (Whiteside et al., 2007; Adams, 2019; Bloomfield et al., 2019; Mendoza and Martone, 2019).
7. **Develop Your Mentoring Style**. We are not always given the opportunity to think about and develop *how* we want to work with students. Thinking back to my undergraduate days, my advisor used her enthusiasm for research to get students interested and engaged in her work. Holmes and Roberts (2019) would classify this as a Mentor-as-a-Makeover-Artist strategy because it gets students interested in one's own ideas. As a faculty member, especially pre-tenure, I gravitated to this approach because I had the expertise and natural enthusiasm to best guide projects. However, students do not have the depth of knowledge, so involving them in this way can be challenging. Since all majors need a thesis to graduate, I also cannot always rely on this strategy. Sometimes, I need to allow students to explore their own ideas, but I need to transform those ideas into something rigorous and publishable. Holmes and Roberts (2019) refer to this as the Mentor-as-a-Sculptor style. In this approach, students take ownership of a project, but the ideas typically fall outside the advisor's area of expertise making it more challenging to mentor. I have found that I am more willing to engage in this mentoring style now that I am post-tenure. Overall, taking time to think critically about how I want to mentor students has helped in the management of the research projects.
8. **Utilize Resources.**
  - **Institutional Resources**. It is important to look at what your institution offers, and see which of those resources can be useful (Dutta et al., 2019; Mickley Steinmetz and Reid, 2019). For instance, I use our project-based curriculum to attract students into the lab, and I encourage students to apply for the summer research fellowship. I also utilize our study abroad program to engage students in publishable cross-cultural research (Skorinko et al., 2015). However, it is important to note that cross-cultural research requires care, sensitivity, and flexibility (Ashdown, 2019; Burns-Cusato and Cusato, 2019; Hill and Karlin, 2019).
  - **The Classroom**. I also use the classroom to engage students in research. Regardless of the topic, I always cover methodologies to provide a foundation for the research we will discuss throughout the term. I also create assignments that incorporate these methods. For instance, students test theories through observational studies or surveys/interviews. While these projects will most likely not be published, they engage students and pique their interest in research opportunities outside of class.
  - **Research Methods/Statistics Courses**. There are a number of different models for teaching Research Methods and Statistics courses (LoSchiavo, 2018; McKelvie and Standing, 2018; Mendoza and Martone, 2019). In my own course, I develop several 2 x 2 between-participant projects that could be publishable. The ideas come from my lab, my colleagues', and my collaborators'. Students rank their preferences, form into teams, and I use the Mentor as a Make-Over Artist Approach (Holmes and Roberts, 2019) to help them take ownership and develop the project. We utilize our participant pool and Amazon's Mechanical Turk for data collection. In the end, students show deep learning and understanding about experimental design and analysis, are excited about their work, and sometimes it is publishable (most recent example: Riemer et al., 2018).
  - **Collaboration**. Like others, I also develop collaborations, research networks, and mentoring opportunities with colleagues at my home institution and other institutions (Bukach et al., 2019; Hammersley et al., 2019). However, all my collaborators know that the work I conduct will involve undergraduate research assistants (and co-authors).
9. **Be Mindful of Your Time**. At each stage in my career, I needed to protect my time, in different ways, to achieve tenure or promotion (Mendoza and Golden, 2019). This special issue provides a number of different strategies to enable efficiency (Stefanucci, 2019) and management of undergraduate co-authors and teams (Adams, 2019; Mendoza and Martone, 2019; Scisco et al., 2019; Wood, 2019). For instance, you can have students in the lab mentor one another (Overman, 2019; Reavis and Thomas, 2019).

## Conclusion

I realize the tremendous beneficial effect my undergraduate research experiences had on my education (Lopatto, 2003; Russell et al., 2007). As others have reported, I learned research was both tedious and eye-opening (Todd et al., 2004; Matthews and Rose, 2018). I also developed skills and a mindset that would not have been possible if I waited. I became intellectually curious, learned to think critically, and found myself asking more questions (Hathaway et al., 2002). For the students I have engaged early on, they are reporting the same outcomes. Thus, in my experience (as an undergraduate and as a faculty member), the benefits of engaging undergraduates in publishable research, especially early on, outweigh the challenges.

## Author Contributions

The author confirms being the sole contributor of this work and has approved it for publication.

### Conflict of Interest Statement

The author declares that the research was conducted in the absence of any commercial or financial relationships that could be construed as a potential conflict of interest.
